# Supplementary material for: Breed and housing system shape jejunal transcriptome and digesta microbiota in German Landrace and Saddleback pigs
Source: Anim Microbiome. 2026 Oct 21;8:111. doi: 10.1186/s42523-026-00652-8 (PMC13723130; doi:10.1186/s42523-026-00652-8)
Supplement: Supplementary file 2 [file 42523_2026_652_MOESM3_ESM.docx]

**Breed and housing system shape jejunal transcriptome and digesta microbiota in German Landrace and Saddleback pigs**

**Hina Wahab Khan^1^, Muhammad Arsalan Iqbal^1^, Henry Reyer^1^, Nares Trakooljul^1^, Frieder Hadlich^1^, Michael Oster^1^, Bjoern Kuhla^1^, Jonas Keiler^2^, Klaus Wimmers^1,3^, Siriluck Ponsuksili^1,*^**

^1^ Research Institute for Farm Animal Biology (FBN), Wilhelm-Stahl-Allee 2, 18196 Dummerstorf, Germany.

^2^ Rostock University Medical Center, Institute of Anatomy, 18057 Rostock, Germany.

^3^ University Rostock, Faculty for Agriculture, Civil, and Environmental Engeneering, 18059 Rostock, Germany.

*Correspondence: Siriluck Ponsuksili

Research Institute for Farm Animal Biology (FBN), Wilhelm-Stahl-Allee 2, 18196 Dummerstorf, Germany

Phone: +49 38208 68703; Fax: +49 38208 68702; e-mail: [ponsuksili@fbn-dummerstorf.de](mailto:ponsuksili@fbn-dummerstorf.de)

Received: date; Accepted: date; Published: date

**Abstract**

**Background**

Sustainable pig farming balances animal welfare, productivity, and social acceptance. Organic systems offer enriched environments for the pigs and represent a model for sustainable production, but require an understanding of how **breed**, intestinal microbiome, and housing interact. In this study, we compared a commercial breed (German Landrace, GL) and a traditional breed (German Saddleback, GS) reared in different housing systems (Conventional, CON; Organic, ECO). Jejunal mucosa transcriptomes (n = 56) and digesta microbiota (n = 100) were collected from growing pigs at 70 days of life; 54 samples overlapped between the two datasets and were used for all integrated host–microbiome analyses. Data from RNA-seq and 16S-seq were evaluated through differential expression analysis, microbial community profiling, and DIABLO-based data integration.

**Results**

Jejunal transcriptome variation was influenced primarily by breed. GL pigs exhibited a profile supporting anabolic pathways, including upregulated oxidative phosphorylation, whereas GS pigs showed a profile focused on lipid metabolism and a shift toward energy storage as an adaptation. In contrast, the housing system was the main driver of microbial composition, with ECO housing enriching fermentative taxa such as *Lactobacillus* and *Campylobacter*, presumably due to outdoor exposure, while CON housing enriched fewer *Lactobacillus* but more *Clostridium*. Breed–Housing combinations analysis revealed distinct adaptive strategies: the modern GL breed responded strongly to housing conditions at both the transcriptional and microbial levels, whereas the traditional GS breed, while also transcriptionally responsive, maintained a more stable microbial community phenotype across environments. Integrated host-microbiome analysis identified a core set of host genes (e.g., *PCSK9* and *SCD*) that were strongly correlated with housing-specific microbes, defining a lipid–inflammation axis.

**Conclusion**

Breed and rearing environment jointly shape the porcine jejunal transcriptome and microbiota through distinct strategies. Together, these data provide molecular insight into how traditional and modern breeds differentially respond and potentially adapt to divergent husbandry systems, with implications for health and productivity.

**Keywords:** Microbiome; housing system; resilience; traditional breeds; biodiversity

1. **Introduction**

Traditional pig breeds such as German Saddleback (GS) are valued for preserving genetic diversity and their suitability to organic and low-input farming systems [1]. In contrast, conventional breeds like German Landrace (GL) have been selected for high fertility, feed efficiency, rapid growth, and lean carcasses [1]. Though GS pigs are less economically efficient due to higher fat content and smaller litter weight compared to GL [2, 3], their elevated intramuscular fat enhances meat flavor and tenderness, making their products highly valued for quality and taste [4]. It is interesting to note that in GS pigs, the expression of most performance traits was not significantly different between conventional and organic husbandry, whereas modern breeds exhibited significant performance losses under organic husbandry conditions [5, 6], indicating a clear genotype-by-environment (G×E) interaction. However, the underlying physiological and molecular mechanisms in the gut, which are crucial for nutrient absorption and overall health in these different breeds and housing systems, remain poorly understood.

Growing consumer demand for higher welfare standards has driven adoption of organic husbandry, characterized by enriched environments such as straw bedding and outdoor access [7]. These systems differ from conventional production in several key management practices, including a later weaning age. Weaning is a well-documented period of stress and growth retardation [8], and its timing is one factor that may differentiate the adaptive challenges posed by each system. Furthermore, outdoor access, while beneficial for welfare, can increase exposure to environmental pathogens  [9, 10]. In the context of organic production with restricted therapeutic antibiotic use, the inherent capacity of different breeds to maintain health and productivity, defined as their physiological resilience, is a critical factor to investigate.

The gut microbiota acts as a dynamic interface between the host and its environment, critically influencing pig health, metabolism, and nutrient absorption [11-13].  Its composition is highly sensitive to perturbation during key developmental stages, like weaning  [14, 15], and is shaped by the interplay of breed and environmental factors. Recent studies have demonstrated that gut microbial composition differs inherently between commercial and local pig breeds, establishing a clear **breed-associated basis** for colonization [16-18]. Moreover, these breed-specific communities respond divergently to environmental changes, such as dietary shifts [19-23], confirming a direct G×E interaction at the gut ecosystem level. Therefore, the gut microbiota constitutes a prime candidate mechanism for investigating how traditional and modern pig breeds adapt to divergent housing systems. We hypothesize that inherent breed differences between GS and GL breeds establish distinct baseline microbial communities, setting the stage for breed-specific responses to environmental change. These genetically influenced host–microbe dynamics are then challenged and reshaped by the contrasting exposures associated with organic (ECO) versus conventional (CON) husbandry, providing a direct means to understand how breed and environmental factors collectively shape key physiological systems underlying productivity and health.

In this study, we used an integrated approach to investigate host–environment–microbiota interactions in the porcine jejunum. We first profiled the jejunal mucosal transcriptome to identify genes differentially expressed by breed, housing system, and their combination, then characterized the jejunal digesta microbiota in terms of alpha/beta diversity and differentially abundant taxa. Finally, we linked host gene expression with microbial abundance, providing a holistic view of the molecular mechanisms underlying host–microbe interactions.

2. **Materials and methods**

**2.1. Ethics statement**

Tissue collection and animal husbandry were in accordance with the guidelines of the German Animal Protection Law. The procedures were approved by the Ethics Committee of the federal state of Mecklenburg-Western Pomerania, Germany (Landesamt für Landwirtschaft, Lebensmittelsicherheit und Fischerei; LALLF M-V 7221.3-1-029/21).

2.2. **Experimental design and animals**

Experimental procedures followed the ARRIVE guidelines, ensuring animal welfare and minimizing pain or distress. The study employed a 2 × 2 factorial design to investigate the impact of breed and housing system on jejunal gene expression and microbial communities. Two housing systems were compared: conventional (CON) and organic (ECO). The housing systems differed in space allowance, flooring, outdoor access, farrowing management, weaning age, and feeding regimes,  as described in detail previously **[24].**

During gestation, CON sows were housed indoors in pens with partially slatted floors and received a commercial diet twice daily. ECO sows were group-housed in straw-bedded pens with continuous outdoor access and were fed an organic, high-fibre diet via an automated feeder station. For farrowing and lactation, CON sows were housed in conventional pens with farrowing crates and were hand-fed a commercial lactation diet, whereas ECO sows farrowed in larger pens with deep straw bedding and outdoor access, receiving an organic lactation diet ad libitum. Piglets were weaned at day 28 in the CON system and at day 42 in the ECO system. All piglets received commercial (CON) or organic (ECO) solid pre-starter diets from day 14 of life ad libitum, followed by respective starter/growing diets. At the day-70 sampling time point, pigs were receiving the corresponding starter/growing diet. The two dietary regimes differed in nutrient composition at every developmental stage. At the day-70 starter/growing stage, the CON diet contained 16.7% crude protein, 3.0% crude fat, 3.8% crude fibre, and 5.0% crude ash, whereas the ECO diet contained 17.2% crude protein, 4.2% crude fat, 4.5% crude fibre, and 5.5% crude ash. Metabolizable energy was 12.8 MJ/kg in CON and 13.0 MJ/kg in ECO. The complete nutrient composition of the diets is provided in **Supplementary Table S1**. The piglets were derived from sows in their first or second parity. Individual birth weights were recorded, and piglets received supplementary feed from the third week of life and were vaccinated one week before weaning, which occurred at either 28 or 42 days of age depending on the husbandry condition. At days 70, one male and one female per litter are slaughtered and sampled. Day 70 was selected to represent the early growing phase, after the immediate post-weaning transition but before the pronounced metabolic and body-composition changes associated with the finishing phase.

An overview of the experimental design is provided in **Figure 1**. At 70 days of age, pigs were stunned by electronarcosis and exsanguinated. A ~15 cm segment of jejunum was collected approximately 4 meters distal to the pylorus. This segment was opened, rinsed with ice-cold 0.9% NaCl, and processed for parallel transcriptomic and microbiota analyses. For transcriptomic profiling, a balanced subset of animals was selected (n = 56; 14 per breed per housing system, comprising 7 females and 7 males per group). The jejunal mucosa was immediately scraped, chopped, snap-frozen in liquid nitrogen, and stored at –80 °C for RNA sequencing. For microbiota analysis, approximately 2 mL of jejunal digesta was collected from the full cohort of 100 animals, comprising 64 GL pigs (CON = 30, ECO = 34) and 36 GS pigs (CON = 15, ECO = 21). Samples were stored at −20 °C until DNA extraction. Of the 56 animals included in the transcriptomic subset, 54 were also represented in the microbiota cohort, thereby enabling integrated analyses at the individual-animal level.

**Figure 1.** Schematic overview of the study design and analytical framework. At day 70 of life, jejunal mucosa and digesta samples were collected from pigs in a 2 × 2 factorial design, comprising two genetic breeds (GL and GS) raised under two housing systems (Conventional, CON; Organic, ECO). Jejunal mucosa from a balanced subset of animals (n = 14 per group; total n = 56) was subjected to RNA sequencing for differential gene expression analysis, while digesta samples from the entire cohort (n = 100) underwent 16S rRNA gene sequencing to profile microbial community composition and differential abundance. The inset Venn diagram illustrates the overlap between the two datasets: 54 samples were common to both transcriptome and microbiome analyses and were used for **cross-omics integration.**

**2.3. Transcriptome library construction and data preprocessing**

For transcriptomic analysis, jejunal mucosa samples from a total of 56 animals were used in a balanced design with equal numbers of female and male animals(n = 14 per breed per housing system). Total RNA was isolated from approximately 30–60 mg of jejunal mucosa tissue using Tri-Reagent (Sigma-Aldrich, Taufkirchen, Germany), in accordance with the manufacturer's instructions. Subsequent DNase treatment and RNA purification were performed using the RNeasy Mini Kit (Qiagen, Germany). RNA concentration was quantified using a Nanodrop ND-2000 spectrophotometer (Thermo Fisher Scientific, Dreieich, Germany). Subsequently, RNA integrity was assessed using an Agilent 2100 Bioanalyzer (Agilent Technologies Inc., California, USA), yielding an average RNA Integrity Number (RIN) of 8.2 ± 0.5, confirming suitability for downstream sequencing. Stranded mRNA sequencing libraries were prepared from the purified total RNA using the Illumina stranded mRNA prep kit (Illumina, San Diego, CA, USA). The quality and size distribution of the final libraries were verified using an Agilent DNA-1000 chip kit on the Bioanalyzer 2100. Paired-end 2x109bp sequencing was carried out at 750 pm using P3 flowcell on an Illumina NextSeq2000 platform at the sequencing core facility of the Research Institute for Farm Animal Biology (FBN) in Dummerstorf, Germany.

Initial quality assessment of the raw sequencing reads was conducted using FastQC (v0.11.9) [25]. Adapter sequences and low-quality bases were removed with Trim Galore (v0.6.10) [26], discarding reads with an average Q-score below 20 and eliminating any residual adapter-like sequences. The resulting high-quality paired-end reads were aligned to the Sscrofa11.1 reference genome (ENSEMBL release 112) using HISAT2 (v2.2.1) [27], achieving a mapping efficiency of approximately 98%. Gene-level quantification was then carried out using HTSeq (v0.11.2) [28].

**2.4. Differential expression analysis**

An initial total of 34,090 genes with raw count data were subjected to filtering to remove lowly expressed genes. Genes with fewer than 5 counts in more than 70% of samples were filtered out, resulting in 14,352 genes retained for differential expression analysis. To control variance across samples, we applied a variance-stabilizing transformation (VST) to the filtered counts by using DESeq2 in R. Subsequently, Principal Component Analysis (PCA) was performed on the VST data to explore global gene expression patterns and visualize sample clustering. To identify and account for latent batch effects and other unmeasured technical confounders, surrogate variable analysis (SVA) was performed using the sva package (version 3.52.0) within the R programming environment [29]. Two surrogate variables (BV1 and BV2) were identified and included in all subsequent DESeq2 models to improve the accuracy of estimating biological effects. Two modeling strategies were employed to systematically investigate transcriptional variation.

(i) Main Effect Model: To identify genes differentially expressed due to the independent contributions of the experimental factors, a main effect model was implemented:

~ breed + housing system + sex + BV1+ BV2

Here, breed represents the comparison between GL and GS, housing system denotes the comparison between CON and ECO, and sex compares M and F.

(ii) Group model (Breed–Housing combinations) Model: To reveal differentially expressed genes (DEGs) in the contrasts of housing system within each breed, such as GL-CON, GL-ECO, GS-CON, and GS-ECO. This approach is particularly useful for assessing gene expression changes within each breed under each housing comparison and for minimizing sample variability. The model was specified as:

~ group + sex + BV1 + BV2.

Differential expression analysis was performed using DESeq2 (version 1.46.0) in R [30]. DEGs were identified using Wald tests, and p-values were adjusted for multiple testing using the Benjamini–Hochberg procedure to control the false discovery rate (FDR). Genes with an adjusted p-value (FDR) < 0.05 were considered statistically significant. Results were visualized using volcano plots generated with the ggplot2 package (version 1.46.0) in R [31].

**2.5. Functional enrichment analysis**

The DEGs from the breed comparisons (GL *vs.* GS) and housing systems (ECO *vs.* CON) were analyzed for Gene Ontology (GO) Biological Processes (BPs) and KEGG (Kyoto Encyclopedia of Genes and Genomes) pathways using the DAVID (version 2024q2) bioinformatics resources [32]. Additionally, the DEGs from four combination groups between breed and housing system, including GL-ECO *vs* GL-CON, GS-ECO *vs* GS-CON, GS-ECO *vs* GL-ECO, and GS-CON *vs* GL-CON, were examined using the ClueGO [version 2.5.1, [33]] and Cluepedia [version 1.5.1, [34]] plug-ins within the Cytoscape environment [version 3.10.2, [35]] for a functionally annotated KEGG pathway network. The parameters for both ClueGO and DAVID analyses included right-sided hypergeometric tests for p-value calculation, Benjamini–Hochberg correction for multiple testing, and Sus scrofa as the reference genome. The KEGG pathways and biological processes with p-values ≤ 0.05 were considered significant.

**2.6. Digesta DNA Extraction and 16S rRNA Gene Sequencing**

Microbial genomic DNA was extracted from 100 mg of jejunal digesta using the DNeasy PowerLyzer PowerSoil Kit (Qiagen, Hilden, Germany). The extraction protocol was optimized by incorporating two additional heating steps at 70°C and 95°C for 10 min each before bead-beating, which improved overall cell lysis efficiency and quality of extracted genetic material. The concentration and purity of the extracted DNA were assessed using a NanoDrop ND-2000 spectrophotometer (Thermo Fisher Scientific, Waltham, MA, USA). The V4 hypervariable region of the 16S rRNA gene was amplified in duplicate using primers 515F and 806R, each tailed with Illumina adapter sequences and unique dual-index barcodes to enable multiplexing. PCR amplification was performed using GoTaq G2 Hot Start polymerase (Promega, Mannheim, Germany) under the following cycling conditions: initial denaturation at 95°C for 2 min, followed by 35 cycles of denaturation (95°C for 30 s), annealing (50°C for 60 s), and extension (72°C for 90 s), with a final extension at 72°C for 10 min to ensure complete amplicon synthesis[36]. Finally, the PCR products were purified and normalized to equal concentrations using the SequalPrep Normalization Plate (Thermo Fisher Scientific). The Sequencing was carried out on the Illumina NextSeq2000 platform (Illumina, San Diego, CA, USA), generating 2x250 bp paired-end reads.

**2.7. Quality control and OTU classification**

Raw 16S rRNA amplicon sequences from jejunal samples of 70-day-old animals were processed and analyzed using the mothur pipeline [version 1.47, [37]], following the standard operating procedure. Sequences were quality-filtered, aligned to the SILVA reference database (release 138), and screened for chimeric reads. Operational taxonomic units (OTUs) were clustered at a 97% sequence similarity threshold. To account for differences in sequencing depth, the OTU abundance and taxonomy tables were rarefied to an even sampling depth across all samples. The resulting dataset was then compiled into a Phyloseq object within the R programming environment, integrating abundance, taxonomy, and sample metadata for subsequent community and diversity analyses.

**2.8. Microbial Diversity and Differential Abundance**

Alpha diversity was quantified using the Shannon diversity index via the Phyloseq (version 1.48.0) package in R [38], capturing both species richness and evenness within samples. Differences in alpha diversity were evaluated using two-way analysis of variance (ANOVA), with breed (GL vs. GS) and housing system (CON vs. ECO) as the two factors. Model assumptions were assessed using the Shapiro–Wilk test for normality of residuals and Levene’s test for homogeneity of variance. Tukey-adjusted pairwise comparisons among the four combined breed and housing groups (GL-CON, GL-ECO, GS-CON, and GS-ECO) were performed using the emmeans package (version 2.0.3). Boxplots were used to visualize the distribution of Shannon diversity according to breed, housing system, and the four combined breed and housing groups. Community composition (beta diversity) was assessed using Bray–Curtis dissimilarities and tested by permutational multivariate analysis of variance (PERMANOVA) using the vegan package (version 2.7.1) in R [39]. To further examine differences among the four combined breed and housing groups, pairwise PERMANOVA analyses were performed separately for each of the following group comparisons: GL-CON vs. GL-ECO, GS-CON vs. GS-ECO, GL-CON vs. GS-CON, and GL-ECO vs. GS-ECO. The proportion of variance explained (R²) by breed, housing system, and the pairwise group comparisons was visualized using bar plots. As a complementary assessment of community dispersion, within-group Bray–Curtis dissimilarities were compared using Wilcoxon rank-sum tests. Because pairwise distance observations are not statistically independent, these comparisons were considered descriptive and complementary. PERMANOVA based on the full Bray–Curtis distance matrix was considered the primary inferential analysis of community-level differences.

Microbial community composition was assessed using stacked bar plots of relative abundances from the complete OTU dataset (16,281 OTUs), generated with ggplot2 [version 3.5.2, [31]], to display the dominant taxa across breed, housing system, and group, illustrating shifts in community composition under different genetic and environmental conditions. Subsequently, OTU counts were aggregated at the genus level (1,299 genera) for differential abundance analysis and filtered to exclude low-abundance genera (counts <3 in more than 50% of samples), thereby minimizing noise from rare taxa. The filtered dataset (100 genera) was analyzed using DESeq2, applying the same modeling framework and surrogate variable approach as outlined for the transcriptomic data (Section 2.3). The genera exhibiting significant differences in relative abundance were identified based on a false discovery rate (FDR) threshold of 0.05.

**2.9. Multivariate integration of jejunum transcriptome and microbiome data**

An integrated multivariate framework was applied to examine associations between the host jejunum transcriptome and microbiome data. A total of 54 samples shared between the 16S rRNA sequencing and mRNA datasets were selected for integration. Variance-stabilized mRNA counts (14,352 genes; see Section 2.3) and genus-level aggregated microbiome abundances (100 genera; see Section 2.7) were utilized. To handle compositionality and excess zeros, microbial counts underwent multiplicative zero replacement and centered log-ratio (CLR) transformation using **zCompositions** (version 1.5.0) and **compositions** (version 2.0.8) R packages. Integration of the datasets and selection of discriminative features were performed using DIABLO (Data Integration Analysis for Biomarker discovery using Latent variable) in mixOmics [version 6.24.0 [40]] with a full design matrix (0.1), balancing inter-block correlations with group discrimination within the R programming environment. The model included two components (ncomp = 2), and the number of features per block (keepX) was optimized via grid search combined with repeated 5-fold cross-validation using the Balanced Error Rate (BER) and a maximum distance criterion to select the optimal parameters. Selected features were visualized using circos plots (correlation cutoff = 0.5) and loading plots, with top variables colored according to the experimental group exhibiting the highest mean abundance or expression. For the DIABLO-selected features, effect sizes and statistical significance were subsequently assessed using group-effect differential expression analyses with DESeq2 (mRNA model: ~ group + sex + BV1 + BV2; microbiome model: ~ Group + sex + SV1 + SV2). The group effect was used to obtain log2 fold-change (log2FC) estimates and Benjamini–Hochberg-adjusted P values (padj) for the selected features. Features with padj < 0.05 were considered statistically significant.

3. **Results**

There were no significant differences in birth weight or body weight at 70 days of age among housing systems, breeds, or sexes. At birth, GS pigs tended to be heavier than GL pigs (1.52 vs. 1.41 kg; *P* = 0.09), and males tended to be heavier than females (1.51 vs. 1.42 kg; *P* = 0.07). However, these differences did not reach statistical significance. Similarly, no significant difference in body weight was observed at 70 days of age between DS and DL pigs (25.52 vs. 24.35 kg; *P* = 0.46). A total of 56 RNA-seq libraries yielded 1.46 billion raw reads, of which 1.40 billion were successfully mapped to the *Sscrofa11.1* reference genome, corresponding to an average mapping rate of 95.9%. Approximately 59.8 million reads (4.1%) remained unmapped. Each library produced an average of 26.1 ± 3.2 million raw reads, of which 25.0 ± 3.1 million were mapped, with mapping efficiencies ranging from 94.0% to 97.2% **(Supplementary Table S2)**.

**3.1. Principal component analysis of main effects and their group effects**

PCA of variance-stabilized transcriptomic data showed substantial overlap among samples, with PC1 and PC2 explaining 43.7% and 9.5% of the variance, respectively. Neither breed (GL vs. GS) nor housing system (CON vs. ECO) showed clear cluster separation **(Figure 2)**.

**Figure 2.** PCA-based visualization of jejunal gene expression patterns. Breeds are represented by different colours (GL = red, GS = dark green), and housing systems are indicated by different shapes (CON = circle, ECO = triangle). Dashed ellipses show the 95% data ellipse for each breed. The x-axis (PC1, 43.7%) captures the largest proportion of variance, while the y-axis (PC2, 9.5%) accounts for additional variance. Individual dots represent animals.

**3.2. Differential expression and functional enrichment analysis of main effects**

Differential gene expression analysis was conducted to evaluate the transcriptional divergence of the main experimental factors: Breed (GS *vs.* GL), system (ECO *vs.* CON), and sex (M *vs.* F). The strongest effect was observed for breed, with 2,489 DEGs, of which 1,876 were upregulated and 613 downregulated in GL compared to GS **(Figure 3A)**. An overrepresentation of metabolic processes, including carboxylic acid, amino acid metabolism, and oxidative phosphorylation, was observed, with most DEGs upregulated in the GL breed. Similarly, genes upregulated in GL were also enriched in cellular organization and transport-related processes, such as actin cytoskeleton organization, active transmembrane transporter activity, and vesicle-mediated transport to the plasma membrane. Genes associated with physiological system processes, such as smooth muscle contraction, endocrine system development, and regulation of fat cell differentiation, exhibited a slightly higher proportion of upregulation in GS compared to GL **(Figure 3B)**. The KEGG pathway analysis further supported these findings, showing coordinated upregulation of metabolic pathways (thermogenesis, AMPK signaling), signaling pathways (adipocytokine, insulin, cellular senescence, adrenergic signaling, AGE-RAGE signaling), and structural/cell cycle pathways (cell cycle, regulation of actin cytoskeleton, tight junction) in GL compared to GS **(Figure 3C)**.

The housing system comparison revealed 1,405 DEGs, with 697 genes upregulated and 708 downregulated in ECO compared to CON, as shown in **Figure 3D**. Gene ontology analysis revealed a significant enrichment of metabolic processes in ECO, including fatty acid biosynthesis, acyl-CoA metabolism, cholesterol transport, and monocarboxylic acid biosynthesis, with most DEGs showing increased expression. In addition, the upregulated genes in ECO were associated with processes related to cellular organization and barrier integrity, such as extracellular matrix organization, external encapsulating structure organization, and cell–substrate junction assembly. In contrast, upregulated genes in CON were predominantly enriched in stress response biological processes, such as responses to reactive oxygen species and response to decreased oxygen levels **(Figure 3E)**. Consistent with these, KEGG pathway enrichment analysis revealed that upregulated genes in ECO were predominantly involved in metabolic regulation (cholesterol metabolism, AMPK and PPAR signaling), nutrient utilization (amino/nucleotide sugar metabolism, and mineral absorption), protein handling, and immune function (Protein processing in the endoplasmic reticulum, and phagosome). Conversely, genes upregulated in the CON group were associated with cellular stress pathways, including FoxO signaling, ribosomal function, and HIF-1 signaling (**Figure 3F).** We found that sex had a minimal effect on gene expression in this study, as only 12 genes were differentially expressed, and these were not enriched for any specific biological pathways. Detailed statistics for all main-effect differentially expressed genes are provided in **Supplementary Table S3**, whereas their functional enrichment, including Gene Ontology terms and KEGG pathways, is summarized in **Supplementary Table S4**.

**Figure 3.** Differential gene expression and functional enrichment by breed and housing system. **(A)** Volcano plot of differentially expressed genes (DEGs) between breeds (GS *vs.* GL). (**B–C)** The bar charts represent the top enriched **(B)** GO Biological Processes and **(C)** KEGG pathways for the breed comparison. **(D)** Volcano plots illustrate the distribution of DEGs for housing (ECO *vs.* CON) contrasts. **(E–F)** The bar charts represent the top enriched **(E)** GO Biological Processes and **(F)** KEGG pathways for housing comparisons. Both volcano plots display genes by log₂ fold change (x-axis) and -log₁₀ adjusted *p*-value (y-axis). For all enrichment bar charts, bar lengths represent the percentage of DEGs associated with each term, and colors indicate the direction of regulation (red = higher in GS or ECO; dark green = higher in GL or CON). Statistical significance is shown as *p < 0.05, **p < 0.01, ***p < 0.001.

**3.3. Differential expression and functional enrichment analysis of breed–housing groups**

Differential gene expression analysis was performed to evaluate the effects of breed–housing combinations on the jejunal transcriptome across the four experimental groups (GL–CON, GL–ECO, GS–CON, GS–ECO). The most pronounced divergence was observed between the GS and GL breeds under organic conditions (GS–ECO *vs.* GL–ECO), comprising 1,935 DEGs, 266 of which were upregulated and 1,669 downregulated in GS–ECO **(Figure 4A)**. The breeds under conventional conditions indicated moderate transcriptomic differences, with GS–CON *vs.* GL–CON showing 374 DEGs, 168 upregulated and 206 downregulated **(Figure 4B)**. The GS exhibited a transcriptomic response to the housing system, with GS–ECO *vs.* GS–CON revealing 351 DEGs, of which 89 were upregulated and 262 downregulated **(Figure 4C)**. The effect of organic housing (ECO) was strongest within the GL breed (GL–ECO *vs.* GL–CON: 857 DEGs; 621 upregulated, 236 downregulated), as shown in **Figure 4D**. Detailed statistics for all combined-effect differentially expressed genes are provided in **Supplementary Table S5**.

Comparative Gene Ontology analysis of DEGs between GS–ECO and GL–ECO revealed a significant upregulation of genes in the GL–ECO group associated with bioenergetic and biosynthetic capacity (amide metabolic process, amide biosynthetic process, organophosphate biosynthetic process, membrane lipid biosynthetic process), coordinated cellular restructuring and genomic replication (plasma membrane organization, organophosphate ester transport, mitochondrion organization, DNA replication), and an adaptive cellular stress response (response to endoplasmic reticulum stress, detoxification). When comparing GS–ECO and GL–ECO, KEGG analysis revealed a similar trend: GL–ECO upregulated genes enriched in metabolic and immunological pathways, such as energy-producing and anabolic pathways (oxidative phosphorylation, AMPK, insulin signaling), structural and lipid-associated processes (muscle cytoskeleton organization, glycerolipid and sphingolipid pathways), and immune activation mechanisms (T cell receptor signaling and complement/coagulation cascades), as shown in **Figure 4E–F**.

Furthermore, Gene Ontology (GO) enrichment analysis comparing GS–CON and GL–CON groups identified clear breed-specific differences in intestinal biological processes. The GL–CON group showed upregulation of pathways related to innate immune activation and mucosal barrier maintenance, including complement activation and glycosaminoglycan metabolism. In contrast, the GS–CON group exhibited higher expression of genes involved in intestinal morphogenesis and lipid transport, such as digestive tract development and long-chain fatty acid transport. The KEGG pathway analysis further supported the differences observed between the two breeds under conventional housing. The GL–CON group showed upregulation of pathways associated with innate immune activation, including the complement and coagulation cascades, whereas the GS–CON group showed upregulation of metabolic pathways related to amino acid and lipid metabolism (arginine and proline metabolism, glycine, serine, and threonine metabolism, and PPAR signaling), as well as peroxisome function and tryptophan metabolism **(Figure 4E–F)**.

Within the GS breed, ontology analysis demonstrated that the GS–ECO group exhibited higher expression of jejunal genes associated with lipid metabolism, including pathways for cholesterol metabolism, fatty acid biosynthesis, lipid homeostasis, and triglyceride metabolism. KEGG pathway analysis further showed that the GS–ECO group was enriched for genes involved in metabolic pathways (PPAR signaling, cholesterol metabolism), absorptive processes (mineral absorption), and immune responses (IL-17, TNF, and Toll-like receptor signaling) in the jejunum **(Figure 4E–F)**.

Gene Ontology analysis revealed that within the GL breed, the comparison of housing systems showed a transcriptional response distinct from that of the GS breed. The GL–ECO group exhibited a coordinated downregulation of core anabolic processes in the jejunum, including translation, cytoplasmic translation, ribosomal small subunit biogenesis, cytoplasmic translational initiation, and triglyceride metabolic process. In contrast, processes related to lipid catabolism, cholesterol handling, and antioxidant defense were upregulated, including cholesterol homeostasis, chylomicron remodeling, and glutathione metabolic process. Analysis of KEGG pathways within the GL breed demonstrated that the GL–ECO group exhibited substantial transcriptional repression of key metabolic and biosynthetic pathways in the jejunum, including ribosome, PPAR signaling, glycolysis/gluconeogenesis, and retinol metabolism. Conversely, the cholesterol metabolism pathway was upregulated in the GS–ECO group **(Figure 4E–F)**. The complete set of enrichment results is provided in **Supplementary Table S6.**

**Figure 4.** Differential gene expression and functional enrichment of group-wise comparisons **(A–D)** Volcano plots showing the number and distribution of DEGs in **(A)** GS–ECO *vs.* GL–ECO, **(B)** GS–CON *vs.* GL–CON, **(C)** GS–ECO *vs.* GS–CON, and **(D)** GL–ECO *vs.* GL–CON. Genes upregulated in each contrast are highlighted in red, whereas downregulated genes are shown in blue. **(E–F)** Pie chart–style dot plots showing enriched gene ontology (Biological Process) terms and KEGG pathways for each pairwise comparison. Each pie’s size denotes the number of genes within a term or pathway, and color proportions (red = upregulated; blue = downregulated). Statistical significance is denoted by asterisks (*p < 0.05, **p < 0.01, ***p < 0.001).

**3.4. Overlap of differentially expressed genes across breed, housing, and their combined groups**

The intersection analysis showed that breed had the strongest overall effect on jejunal gene expression, with 2,489 DEGs between GS and GL pigs, followed by the breed comparison under ECO conditions (GS-ECO vs. GL-ECO; 1,935 DEGs) and the overall housing comparison (ECO vs. CON; 1,405 DEGs) (**Figure 5**). In contrast, fewer DEGs were detected for GS-CON vs. GL-CON (374) and for the within-breed housing comparison in GS pigs (GS-ECO vs. GS-CON; 351). The largest overlap occurred between the overall breed comparison and GS-ECO vs. GL-ECO, with 1,467 shared DEGs, indicating that a substantial proportion of the breed-associated transcriptional differences was also evident under ECO conditions. Housing-related overlap was more moderate, including 514 DEGs shared between the overall housing comparison and GL-ECO vs. GL-CON. Higher-order intersections contained progressively fewer genes, suggesting that most transcriptional responses were comparison-specific rather than common across all breed and housing contrasts. Only one gene, *MT3*, was shared across all six comparisons. Overall, the overlap pattern indicates that breed-related transcriptional differences were more pronounced than housing-related differences, particularly under ECO conditions, whereas relatively few genes were consistently affected across all comparisons.

**Figure 5.** Overlapping DEGs across breed, housing, and their combined groups.UpSet plot of significant differentially expressed genes (DEGs; FDR < 0.05) across six comparisons: breed (GS vs. GL), housing system (ECO vs. CON), and four breed and housing combined group comparisons (GL-ECO vs. GL-CON, GS-ECO vs. GS-CON, GS-CON vs. GL-CON, GS-ECO vs. GL-ECO). Horizontal bars show total DEGs per comparison; vertical bars show the size of each intersection. The dot matrix indicates which comparisons contribute to each intersection: a single dot denotes DEGs specific to that comparison, whereas connected dots denote DEGs shared between the corresponding comparisons.

**3.5. Microbial community composition under main effects and breed–housing combinations**

The relative abundance of microorganisms at the phylum, family, and genus levels revealed significant effects of both breeds and housing system on gut microbiota composition. Bacillota dominated the microbial community across all conditions, accounting for 75.2–86.3% of the relative abundance. Campylobacterota was the second most abundant phylum (6.0–17.4%), whereas all remaining taxa together represented <8% of the community **(Figure 6A–I)**. The breed had a significant impact on microbial composition, with GS breeds showing a higher relative abundance of Bacillota (84.2%) than GL breeds (80.4%). In contrast, GL breeds exhibited higher abundances of Campylobacterota (12.1% vs. 9.2%) and Actinomycetota (4.9% vs. 2.3%) compared to GS breeds. The phylum-level differences persisted throughout the taxonomic hierarchy, with identical patterns observed at the family and genus levels **(Figure 6A–C)**. The housing environment exerted a pronounced effect on microbial community composition. Organic housing exhibited markedly higher Campylobacterota abundance (ECO, 15.1%) relative to conventional housing (CON, 6.1%), corresponding to a 2.5-fold increase. Meanwhile, Bacillota showed a modest decrease in ECO housing (79.2% vs. 84.9% in CON), and Actinomycetota remained unchanged, displaying stable proportions across both housing conditions (3.9–4.0%) (**Figure 6D–F**).

Under breed–housing combinations, GL breeds were particularly sensitive to environmental changes. In GL, the housing system triggered a substantial decrease in *Bacillota* (from 86.3% in CON to 75.2% in ECO) and a corresponding 2.8-fold increase in *Campylobacterota* (from 6.2% CON to 17.4% ECO). *Actinomycetota* also increased slightly (from 4.7% in CON to 5.2% in ECO), whereas *Pseudomonadota* and *Bacteroidota* showed moderate decreases. Other minor phyla, including *Methanobacteriota, Verrucomicrobiota, Fusobacteriota*, and *Spirochaetota*, remained relatively low (<0.2%) across housing systems. In contrast, GS breeds maintained remarkable *Bacillota* stability across housing systems (from 81.9% in CON to 85.8% in ECO), with more moderate *Campylobacterota* increases (from 6.0% in CON to 11.5% in ECO). Pseudomonadota decreased sharply in GS under ECO housing (from 7.46% in CON to 0.15% in ECO. Minor phyla, including *Bacteroidota, Methanobacteriota, Verrucomicrobiota, Fusobacteriota*, and *Spirochaetota*, remained low, with slight reductions under ECO housing (**Figure 6G–I)**.

**Figure 6.** Taxonomic composition of jejunal digesta microbiota across main and interactive effects. Stacked bar plots show the mean relative abundance of bacterial taxa at the (A, D, G) phylum, (B, E, H) family, and (C, F, I) genus levels for each experimental group. **(A–C)** Breed **(D–F)** Housing system **(G–I) Breed-Housing groups.**

**3.6. Jejunal digesta microbiota diversity across breeds, housing, and their combination**

Principal Coordinate Analysis (PCoA) of Bray–Curtis dissimilarity demonstrated distinct clustering of jejunal microbial communities, with separation primarily associated with housing system **(Figure 7A).** PERMANOVA on the full Bray–Curtis distance matrix identified **housing system as the sole significant factor** associated with microbial composition, whereas breed explained only a small and non-significant proportion of variance **(Figure 7B).**Two-way ANOVA of Shannon diversity revealed no significant effect of breed or housing system **(Figure 7C–D)**. As a complementary dispersion analysis, within-group pairwise Bray–Curtis distances differed significantly by breed (Wilcoxon p = 4.78 × 10⁻⁵; **Figure 7E**) and by housing system (Wilcoxon p = 8.14 × 10⁻¹⁴; **Figure 7F**).

**Figure 7.** Alpha and beta diversity of jejunal digesta microbiota by breed and housing system (full cohort, n = 100). **(A)** PCoA based on Bray–Curtis dissimilarity (PCoA1 = 28.3%, PCoA2 = 14.3%). GL samples are shown in red and GS samples in dark green; housing systems are indicated by shape, with circles representing CON and triangles representing ECO. Dashed ellipses show the 95% data ellipse for each breed. **(B)** PERMANOVA showing the proportion of variance (R²) explained by breed and housing system. **(C–D)** Boxplots of the Shannon index illustrating alpha diversity by **(C)** breed and **(D)** housing system. P*-*values were derived from two-way ANOVA. **(E–F)** Boxplots of within-group pairwise Bray–Curtis dissimilarity by **(E)** breed and **(F)** housing system (complementary dispersion analysis); P*-*values were derived from Wilcoxon rank-sum tests. ns = non-significant; P*-*values < 0.05 were considered statistically significant.

Principal coordinates analysis (PCoA) of Bray–Curtis dissimilarities showed separation of microbial community structures according to the four combined breed and housing groups (GL-CON, GL-ECO, GS-CON, GS-ECO; **Figure 8A**). Four pairwise PERMANOVA models confirmed that housing significantly altered community composition within both breeds, whereas breed contrasts within each housing system remained non-significant **(Figure 8B)**. Tukey-adjusted post-hoc comparisons between the four groups following the two-way ANOVA were all non-significant **(Figure 8C).** In the four-group dispersion analysis, housing significantly altered community dispersion within the GL breed (GL-CON vs GL-ECO: Wilcoxon p = 8.93 × 10⁻⁶) and within the GS breed (GS-CON vs GS-ECO: Wilcoxon p = 1.22 × 10⁻³), whereas breed had no significant effect on dispersion within either housing system (GL-CON vs GS-CON: p = 0.080; GL-ECO vs GS-ECO: p = 0.534; **Figure 8D**).

**Figure 8.** Interactive effects of breed and housing on jejunal microbiota diversity (full cohort, n = 100). **(A)** PCoA based on Bray–Curtis dissimilarity by the four combined breed and housing groups (GL-CON, GL-ECO, GS-CON, GS-ECO; PCoA1 = 28.3%, PCoA2 = 14.3%). Dashed ellipses show the 95% data ellipse for each group. **(B)** Proportion of variance (R²) explained by PERMANOVA for four pairwise comparisons between the combined breed and housing groups. **(C)** Boxplot of the Shannon index illustrating alpha diversity by group; P-values were derived from Tukey-adjusted post-hoc comparisons following the two-way ANOVA. **(D)** Boxplot of within-group pairwise Bray–Curtis dissimilarity (complementary dispersion analysis); P*-*values were derived from pairwise Wilcoxon rank-sum tests. ns = non-significant; P*-*values < 0.05 were considered statistically significant.

The same analyses were repeated in the 54 paired transcriptome–microbiome samples, and the direction and significance of all effects were consistent with the full cohort. Full statistics are provided in **Supplementary Table S7**.

**3.7. Differential abundance of jejunal microbial genera under main effects and group combination**

Differential abundance analysis revealed significant breed-associated differences in jejunal microbiota, with 11 genera showing distinct abundance patterns between GS *vs.* GL. The majority of differentially abundant genera (8 of 11) exhibited low abundance in GS breeds, most notably *Staphylococcus*, *Bifidobacterium*, and *Veillonella*. Conversely, only three genera were significantly higher in abundance in GS breeds, including *Intestinibacter*, *Actinobacillus,* and *Lachnospiraceae-UCG-007* **(Figure 9A–B)**. The housing system demonstrated a more pronounced effect on microbial composition than host breed, with 55 genera showing differences in abundance, including 9 genera with higher abundance in organic (ECO) housing and 46 genera with higher abundance in CON housing **(Figure 9A)**. ECO housing was characterized by a strong enrichment of a smaller set of taxa, most notably *Campylobacter, Pseudoscardovia,* and *Olsenella*. In contrast, CON housing supported a greater abundance of a wide and diverse range of genera, including *Sharpea, Sarcina, Actinobacillus, Streptococcus,* and *Staphylococcus*, with fewer genera exhibiting lower abundance **(Figure 9B).** Differential abundance analysis of breed–housing combinations demonstrated that the GL breed was highly responsive to housing, with 41 differentially abundant taxa between GL-ECO and GL-CON (8 higher in ECO, 33 higher in CON). The GS breed showed a similar response, with 37 differentially abundant taxa between GS-ECO and GS-CON (4 higher in ECO, 33 higher in CON). Between-breed comparisons within the same housing system indicated slightly more differences in conventional housing (6 taxa) than in organic housing (4 taxa) **(Figure 9A)**. In the GL–ECO *vs*. GL–CON comparison, the most substantially increased genera in GL–ECO included ***Pseudoscardovia****,* ***Campylobacter****,* ***Olsenella****,* ***Aminicella***, and ***Mycoplasmataceae–unclassified***, whereas GL–CON was characterized by higher abundances of ***Sharpea****,* ***Sarcina****,* ***Rothia****,* ***Veillonella****,* and ***Streptococcus* (Figure 9C)**. The comparison between GS–ECO and GS–CON revealed a distinct microbial shift in response to the housing environment. The jejunal microbiota of GS breeds under ECO conditions was significantly enriched with Campylobacter, Mycoplasmataceae–unclassified, Limosilactobacillus, and an unclassified Bacillota genus. In contrast, the CON environment in GS breeds selected for a consortium of taxa, including Pasteurellaceae–unclassified, Actinobacillus, Sarcina, Jeotgalicoccus, and Acetitomaculum **(Figure 9C)**. In breed comparisons within housing systems, GS–CON *vs*. GL–CON revealed a higher abundance of Pseudoscardovia and Actinobacillus in GS breeds. For GS–ECO *vs.* GL–ECO, the GS breed demonstrated increased Intestinibacter and *Lachnospiraceae_UCG-007*, while the GL breed maintained higher Olsenella abundance **(Figure 9C)**. The full differential abundance statistics for all comparisons can be found in the **Supplementary Table S8**.

**Figure 9.** Differentially abundant microbial taxa across breed, housing, and their combined group effects. **(A)** Volcano bar plot showing the number and distribution of significantly differentially abundant bacterial genera across the main effects of breed (GS *vs.* GL), housing system (ECO *vs.* CON), and their combined effect. The x-axis represents the pairwise comparison groups, while the y-axis indicates the number of differentially abundant genera. Colors denote abundance direction: red indicates higher abundance, dark green indicates lower abundance under main effects, and blue indicates lower abundance under the combined effect. Dot plots illustrating differential abundance patterns: **(B)** Main effects of breed and housing, and **(C)** their combined group effect. Each point represents a bacterial genus, with color indicating the direction of log2 fold change (red for positive log2FC, dark green for negative log2FC in main effects, and blue for negative log2FC in combined effects). Dot size represents the magnitude of the absolute fold change (|log2FC|). Statistical significance is denoted by asterisks (*p < 0.05, **p < 0.01, ***p < 0.001).

**3.8. Multi-Omics integration identified distinct molecular and microbial signatures**

The DIABLO model identified robust signatures that clearly discriminated GS–ECO, GS–CON, GL–ECO, and GL–CON along the first two latent components. Component 1 consisted of 15 discriminative features, including five mRNAs and ten microbial taxa. Within the mRNA block, ***PCSK9*** was the dominant feature characterizing GS–ECO, whereas ***ENSSSCG00000014075*** and ***EIF3H*** were associated with GS–CON, and ***SCD*** and ***APOA4*** contributed to GL–ECO **(Figure 10A)**. The microbial block exhibited similar patterns: Mycoplasmataceae-unclassified, Lactobacillus, Limosilactobacillus, Bacteria-unclassified, Lactobacillaceae-unclassified, Helicobacter, and Campylobacter were associated with GS–ECO and GL–ECO, whereas Ruminococcus, Rothia, and Erysipelotrichaceae-insertae-sedis-genus corresponded to GS–CON and GL–CON **(Figure 10B)**. Component 2 consisted of 15 discriminative features, including 10 mRNAs and 5 microbial taxa. In the mRNA block, *SEPTIN3* and *SGK2* were associated with GL–CON, whereas *PLXNA2*, *SH3RF2*, *ENSSSCG00000038048*, *ENSSSCG00000054633*, and *ATP8* contributed to GL–ECO, and *HEBP1* and *ENSSSCG00000049480* were linked to GL–CON. *TBC1D30* was associated with GS–CON **(Figure 10C)**. The microbial block exhibited similar patterns: *Bifidobacterium* characterized GL–CON, *Facklamia* was associated with GL–ECO, and *Actinobacillus* and *Peptostreptococcaceae-unclassified* were linked to GS–CON, while *Jeotgalicoccus* contributed to GL–CON **(Figure 10D)**.

Furthermore, the model identified the most discriminative and correlated features, resulting in a final set of 30 mRNA transcripts and 15 microbial taxa that contributed most to the associations between the two datasets across the first two components. The correlation among these selected features is visualized in the Circos plot, which depicts all pairwise correlations with |r| ≥ 0.5. Interestingly, all 30 DIABLO-selected genes and 11 of the 15 selected microbial taxa were significantly differentially expressed or abundant in at least one group contrast (FDR < 0.05; **Supplementary Table S9**).

Among the selected features, *PCSK9* showed the strongest positive correlations across the dataset. Its expression was strongly correlated with several bacterial taxa, including *Mycoplasmataceae-unclassified* (r = 0.858), *Campylobacter* (r = 0.823), *Lactobacillus* (r = 0.737), *Bacteria-unclassified* (r = 0.722), *Limosilactobacillus* (r = 0.711), and *Helicobacter* (r = 0.631). In contrast, *PCSK9* was moderately negatively correlated with *Ruminococcus* (r = -0.575). Simultaneously, the genus *Bifidobacterium* was identified as a prominent microbial taxon, strongly correlated with several mRNAs, including PLXNA2 (r = 0.737), SH3RF2 (r = 0.718), SEPTIN3 (r = 0.685), ENSSSCG00000038048 (r = 0.697), and ELOVL1 (r = 0.666). *Bifidobacterium* exhibited strong negative correlations with CPM (r = -0.660), TBC1D30 (r = -0.652), PDZD9 (r = -0.619), and SNAPC3 (r = -0.587). Additional notable microbial–gene correlations included *Campylobacter* and *SCD* (r = 0.649), *Lactobacillus* and SCD (r = 0.596), and *Mycoplasmataceae-unclassified* and *SCD* (r = 0.671). *Facklamia* showed positive correlations with *PLXNA2* (r = 0.643) and *HEBP1* (r = 0.638) and negative correlations with *TBC1D30* (r = -0.569) and *SNAPC3* (r = -0.613) as shown in **Figure 10E**.

**Figure 10.** Discriminative molecular and microbial signatures across groups. Loading plots of mRNA **(A–C)** and microbial features **(B–D)** highlight discriminative signatures across groups along the first two latent components. Groups are color-coded as GL–CON (cyan), GL–ECO (purple), GS–CON (orange), and GS–ECO (brown). **(E)** Circos plot visualizing correlations between the mRNA and microbial features selected by the DIABLO model across the first two latent components. The mRNA feature block is shown with green dotted lines, and the microbiome feature block with yellow dotted lines. Red lines indicate positive correlations, and blue lines indicate negative correlations. Colored lines indicate group-specific feature expression: GL–CON (cyan), GL–ECO (purple), GS–CON (orange), and GS–ECO (gray). Correlations with ∣r∣ ≥ 0.5 are included. The displayed features are those selected by the sparse DIABLO model as key discriminators between groups.

4. **Discussion**

This study utilized an integrated approach to elucidate how breed (GL vs. GS) and housing environment (CON vs. ECO) interact to shape the jejunal mucosal transcriptome and the digesta microbiota in pigs. Our findings reveal a distinct contrast: the jejunal mucosal transcriptome is predominantly shaped by **breed,** whereas the jejunal digesta microbiota composition is primarily shaped by the housing environment. To disentangle these contributions, we modeled breed and housing both as main effects and via a four-level group factor (GL-CON, GL-ECO, GS-CON, GS-ECO), enabling contrasts that revealed genotype-specific environmental responses and environment-specific genetic effects in both jejunal gene expression and digesta microbiota.

The most substantial transcriptional differences in our study were breed-driven, with 2,489 genes differentially expressed in the jejunal mucosa between GL and GS pigs. Functional enrichment revealed two divergent strategies for jejunal physiology. The GL breed exhibited a transcriptional profile characterized by enhanced expression of pathways related to oxidative phosphorylation, carboxylic-acid metabolism, mucosal integrity, and nutrient sensing, suggesting differences in mitochondrial activity, nutrient processing, and epithelial maintenance. This was characterized by a coordinated upregulation of genes involved in oxidative phosphorylation and carboxylic-acid metabolism, reflecting elevated mitochondrial energy production likely required to meet the substantial ATP demands of active nutrient transport in enterocytes. This pattern is consistent with established jejunal physiology, where differentiated villus enterocytes rely heavily on mitochondrial oxidative phosphorylation to drive nutrient uptake, first-pass amino-acid catabolism, and maintenance of epithelial barrier function [41-43], and is supported by a study in weaned pigs showing that enhanced mitochondrial function correlates with increased mucosal growth and absorptive [44]. The GL jejunum showed upregulation of pathways related to mucosal integrity and remodeling, indicating enhanced enterocyte turnover, structural plasticity, and barrier function, suggesting differences in epithelial maintenance and nutrient processing [45]. The upregulation of AMPK and insulin signaling pathways in the GL jejunum reflects a transcriptional program for fine-tuned nutrient sensing and allocation, consistent with enhanced intestinal transport, epithelial integrity, and mitochondrial function [44, 46]. In contrast, the GS jejunum showed greater representation of pathways related to lipid metabolism, long-chain fatty-acid transport, and structural development, suggesting a distinct metabolic program involving lipid processing and energy allocation.

Local breeds have been shown to display higher expression of key lipogenic and lipid transport genes in muscle at slaughter compared to modern commercial breeds [47]. In suckling piglets, these phenotypic differences in lipid metabolism pathways may not yet be evident, but they are reflected in plasma total protein levels, which were higher in GS compared to GL [48]. This further emphasizes differences in energy storage adaptations in traditional pig breeds. A recent multi-omics study reported breed-specific gut microbiota and metabolic profiles linked to fat deposition via gut-derived metabolites and lipid biosynthesis pathways [49]. Consistent with this, the GS jejunum in our study showed upregulation of genes involved in adipocyte differentiation and lipid metabolism, particularly under ECO conditions, where fatty acid synthesis and cholesterol metabolism pathways were enriched. These findings suggest that GS pigs may exhibit a distinct transcriptional program related to lipid metabolism and energy allocation. However, the present study did not directly measure lipid deposition or energy-storage phenotypes, and therefore these molecular differences should not be interpreted as direct evidence of enhanced fat deposition. The breed-related transcriptional differences observed in the jejunal mucosa are also broadly consistent with previously reported haematological differences between GL and GS pigs, based partly on overlapping animals from the same experimental population [24]. In particular, the higher haemoglobin-loading capacity observed in GL was previously discussed in relation to the metabolic demands associated with growth and performance, which is conceptually compatible with the enrichment of energy-metabolism-related pathways observed in the jejunal mucosa of GL pigs in the present study, despite the absence of significant differences in body weight between GL and GS pigs [24].

In addition to transcriptional differences, individual microbial taxa in the jejunal digesta showed breed-associated differences in relative abundance, corresponding with the breeds' distinct metabolic strategies. However, at the community level, breed had only a small and non-significant effect on overall microbial composition.  In GS pigs, the abundance of Intestinibacter and Lachnospiraceae-UCG-007. *Lachnospiraceae UCG‑007* belongs to the family *Lachnospiraceae*, which is well-characterized as carbohydrate-fermenting and short‑chain fatty acid (SCFA) producing [50, 51]. Certain *Lachnospiraceae* isolates have been shown to generate not only classical SCFAs (acetate, propionate, butyrate) but also longer-chain fatty acids such as valerate and caproate via the reverse β-oxidation pathway [52]. Notably, Lachnospiraceae UCG‑007 abundance has been positively correlated with lipid-related metabolites in pigs [53], suggesting a gut microbiota with potentially enhanced fermentative and metabolite-production capacity. Given the established roles of SCFAs and other microbial metabolites in energy salvage, lipid metabolism, and adipogenesis, this compositional shift may contribute to the enhanced energy retention and fat deposition observed in GS pigs.

In contrast, the GL showed enrichment of several bacterial genera, including *Streptococcus*, *Bifidobacterium*, *Veillonella*, *Erysipelotrichaceae UCG‑002*, and *Staphylococcus*. Among these, *Veillonella* and *Bifidobacterium* are most relevant with respect to carbohydrate fermentation and potential microbial cross‑feeding in porcine gut ecology. *Veillonella spp.*, commonly observed in pig gut microbiota, including in suckling piglets and during early developmental stages, are recognized lactate‑utilizing bacteria that, under appropriate conditions, can convert lactate into SCFAs such as propionate and acetate [54]. Furthermore, lactic acid bacteria and bifidobacteria isolated from the porcine gut are established primary fermenters of dietary carbohydrates [55]. These observations suggest that GL pigs harbor a gut microbiota favorable for rapid carbohydrate fermentation and lactate/SCFA production, consistent with their transcriptional signatures of enhanced mitochondrial activity and nutrient uptake, which may support growth and metabolic performance.

Our functional enrichment analysis of DEGs for housing (ECO vs. CON) showed that pigs in ECO upregulated lipid metabolism and mucosal remodeling pathways, while downregulating cellular stress pathways. In ECO pigs, fatty acid biosynthesis, acyl-CoA metabolism, cholesterol transport, and monocarboxylic acid biosynthesis were significantly enriched, suggesting more efficient jejunal lipid absorption and processing, consistent with reports of enhanced lipid metabolism in outdoor or organic systems [56-58]. Another study demonstrates that SCFAs influence lipid partitioning by inducing the LPL inhibitor *ANGPTL4* and increasing hepatic AMPK activation [59]. Alongside the observed metabolic changes, ECO pigs exhibited coordinated upregulation of processes associated with intestinal mucosal remodeling, including extracellular matrix organization, external encapsulating structure formation, and cell–substrate junction assembly. These results are in line with previous reports that environmental enrichment or outdoor rearing can influence intestinal gene expression, immune status, and gut microbiota composition, thereby supporting barrier integrity and mucosal resilience [60, 61]. In contrast, CON pigs showed upregulation of pathways related to oxidative stress and hypoxia, including HIF-1 and FoxO signaling, indicating greater cellular stress responses. The underlying causes are unclear but may reflect differences in management, environmental load, or microbiome composition.

Importantly, breed–housing combinations showed that ECO effects were not uniform but reflected distinct, breed-specific adaptive strategies. In GL pigs, ECO housing (GL–ECO vs. GL–CON) was associated with strong enrichment of lipid metabolism pathways, consistent with reports that gut-derived SCFAs co-activate PPAR signaling to enhance intestinal lipid absorption [57]. Also, the enrichment of pro-inflammatory immune pathways (e.g., IL-17, TNF, Toll-like receptor signaling) indicates a state of active immune engagement alongside metabolic reprogramming, consistent with previous studies showing that pigs exposed to more complex microbial environments exhibit elevated IL-17-producing cells and enhanced TNF-α responsiveness [61, 62]. In contrast, GS pigs under ECO conditions (GS–ECO vs. GS–CON) exhibited downregulation of energy-intensive processes, such as translation, ribosome biogenesis, and glycolysis, indicating a metabolic shift within the jejunal absorptive epithelium. This shift reduced cell turnover and nutrient processing, enabling energy conservation and maintaining barrier integrity to conserve energy and maintain barrier integrity. Such a response is consistent with the integrated stress response, wherein diverse cell types and organisms similarly suppress anabolic processes to conserve energy and promote survival during challenging conditions [63, 64]. Under conventional housing conditions, each breed exhibited unique inherent traits, reflecting their genetic divergence. The GS breed in conventional housing (GS-CON) showed enrichment of pathways related to digestive tract morphogenesis and long-chain fatty acid transport, suggesting inherent traits favoring intestinal growth and lipid absorption, consistent with earlier findings on breed-specific small intestinal transporter expression [65, 66]. In contrast, GL‑CON pigs showed upregulation of immune-activation pathways, suggesting a baseline state of heightened barrier defense. This aligns with previous studies reporting that intensive or high-density housing in pigs is associated with elevated immune-related transcriptomic signatures, reflecting increased immunological activity under conventional management conditions [55].

The distinct host transcriptomic profile in ECO pigs is strongly supported by concurrent shifts in the jejunal microbiota, revealing a coherent host-microbe adaptation to the organic environment. The microbiota of ECO pigs was enriched in fermentative genera, including Lactobacillus, whereas GL-ECO pigs showed a specific enrichment of Limosilactobacillus, while species of both genera are known contributors to SCFA-mediated energy metabolism. For instance, higher levels of *Lactobacillus frumenti* in piglets have been correlated with increased hepatic fatty acid β-oxidation, although a causal relationship has not been established [67]. *Limosilactobacillus* are core, beneficial gut commensals, with previous studies showing that dietary supplementation of *Limosilactobacillus reuteri* reduces pathogen load in weaning pigs [68]. Our study found that Limosilactobacillus was more abundant in the GS–ECO group. Organic (ECO) housing enriched a relatively small but distinct set of taxa, notably *Campylobacter, Pseudoscardovia*, and *Olsenella*. The enrichment of Olsenella, a genus associated with elevated acetate and lactate production [69], was primarily observed in the GL breed raised in the ECO environment (GL-ECO). The genus *Pseudoscardovia*, which encompasses the species *P. radai*, showed higher abundance under ECO conditions, with the strongest enrichment observed in GL-ECO pigs. While *P. radai* has been associated with pasture-raised systems [58], it is unclear whether this association extends to the entire genus. The association of *Pseudoscardovia* and *Olsenella* with the intestines of wild or pasture-raised pigs suggests that ECO conditions partially restore a ‘wild-type’ microbiota profile. However, the concurrent enrichment of *Campylobacter* aligns with reports of high *Campylobacter coli* prevalence in pigs with outdoor access and organic production, underlining a potential trade-off between naturalized microbiota and zoonotic risk [70-72]. Pigs under CON housing showed shifts in microbial composition, including increased abundance of taxa associated with immune activation, and upregulation of pathways linked to inflammatory responses, compared with ECO pigs. Genera enriched under conventional housing in both breeds, including Actinobacillus, Streptococcus, Pasteurellaceae, and Rothia, contain species that can be pathogenic. Staphylococcus and Escherichia–Shigella were particularly enriched in GL-CON pigs. However, many members of these genera are normal commensals of mucosal surfaces. These taxa have well-established roles in porcine health challenges; for instance, *Actinobacillus* species can cause septicemia or pneumonia under stressful conditions, such as during weaning or transport [73], while *Streptococcus* *suis* is associated with systemic disease, inflammation, and muscle damage [58, 74, 75]. Similarly, elevated *Pasteurellaceae* *and Escherichia–Shigella* have been associated with gut perturbation and mucosal damage [76, 77]. Meanwhile, livestock-associated *Staphylococcus* *aureus* (MRSA) is frequently detected in conventional pig farms, with its prevalence rising with farm size and production intensity [78].

Finally, the integration of host transcripts and gut microbiome data allows us to suggest a potential link between housing-specific microbial changes and host transcriptional responses. Gut microbes are recognized as key regulators of host metabolism, particularly lipid metabolism and lipogenesis [79]. One important mechanism involves bile acids (BAs), which are synthesized from cholesterol in the liver and subsequently chemically modified by the gut microbiota in the intestine [80]. These microbial transformations of BAs influence their signaling properties through receptors such as FXR and TGR5, thereby affecting host lipid synthesis, energy balance, and overall metabolic homeostasis. There is clear experimental evidence showing that changes in gut microbiota composition can directly drive shifts in host gene transcription, including nutrient handling and barrier-related genes [81].

*PCSK9* plays a significant role in cholesterol transport in intestinal epithelial cells via degradation of low-density lipoprotein receptor (LDLR) [82]. In our study, *PCSK9* expression was highly positively correlated with both *Campylobacter/Mycoplasma* and *Lactobacillus/Limosilactobacillus*, particularly in ECO pigs of both breeds, suggesting a link between microbial composition and host lipid regulation. Among other discriminative features, transcripts involved in lipid metabolism and lipogenesis, including *SCD, APOA4*, and *ELOVL1*, showed strong correlations with Lactobacillus, a genus enriched in ECO pigs and known for bile-salt–hydrolase activity. Pigs are recognized as reservoirs of *Campylobacter* [83], with high carriage rates reported in both organic/free-range and conventional systems [70, 71]. However, Organic systems, with lower antimicrobial use, may promote expansion of facultative taxa such as Lactobacillus and Limosilactobacillus [84]. Mechanistically, Lactobacillus and Limosilactobacillus are bile-tolerant and often carry bile-salt hydrolases [85], Campylobacter tolerates bile via the CmeABC efflux [86], and *mycoplasma* lipoproteins activate TLR2-driven inflammation [87]. The observed positive correlations of *PCSK9* with these taxa suggest that microbial bile-modifying and inflammatory signals converge on a shared lipid/inflammation axis, linking microbiota composition to host transcriptional regulation in ECO pigs.

**A limitation of the present study is that the husbandry systems differed simultaneously in several factors, including space allowance, flooring, feeding, and weaning age. Consequently, observed CON–ECO differences should be interpreted as system-level effects, as the contributions of individual management factors cannot be disentangled. Dietary differences may also have contributed to the observed transcriptomic and microbial profiles. In addition, variation in bulk jejunal gene expression may reflect differences in cellular composition, physiological and nutritional status, stress responses, and microbiome-associated processes. Thus, the identified molecular patterns should be regarded as integrated signatures associated with breed and husbandry system rather than as evidence of specific causal mechanisms.**

**Conclusion**

In this study, genetic background was associated with the largest differences in the jejunal host transcriptome of piglets, including expression signatures consistent with divergent metabolic programs, more anabolic-related expression patterns in GL and more energy-conserving or maintenance-related patterns in GS. In contrast, the housing systems mainly influenced the jejunal microbiota: organic housing was associated with more fermentative microbial communities, whereas conventional housing showed higher relative abundance of taxa previously linked to pro-inflammatory states. GL pigs showed housing-dependent enrichment of metabolic and immune adaptation pathways, while the traditional GS breed maintained a more stable transcriptional state across both environments. Integrated analyses combining host transcriptomics and microbial features identified statistically supported co-variation patterns linking specific microbial genera (e.g., *Lactobacillus, Campylobacter*) with host lipid- and inflammation-related genes (e.g., *PCSK9, SCD*). However, multi-omics integration captures correlation rather than mechanistic directionality. Therefore, follow-up studies using larger G×E cohorts, additional time points, and targeted functional validation, ideally complemented by higher-resolution microbiome and functional profiling approaches (e.g., shotgun metagenomics, metatranscriptomics, and metabolomics), will be required to assess causality and to connect these molecular signatures to measurable performance or health outcomes.

**Data Availability**

No special codes were used in this study.

Expression data (raw FASTQ files and associated sample metadata) are available in the EMBL-EBI ArrayExpress database under accession E-MTAB-16315. The 16S rRNA gene sequencing data are available in the Sequence Read Archive under the bioproject PRJNA1370827.

For reviewer access links:

16S rRNA (PRJNA1370827): <https://dataview.ncbi.nlm.nih.gov/object/PRJNA1370827?reviewer=r97i15msa23da4c0qcmmu5go79>

Expression data (E-MTAB-16315): <https://www.ebi.ac.uk/biostudies/ArrayExpress/studies/E-MTAB-16315?key=d850dcb6-db4d-4e47-b002-b667f168d1e8>

**Abbreviations**

***ANOVA***: Analysis of variance
***ASV***: Amplicon sequence variant
***BV***: Batch variable / Surrogate variable from surrogate variable analysis
***CLR***: Centered log-ratio transformation
***CON***: Conventional housing system
***DEGs***: Differentially expressed genes
***DIABLO***: Data Integration Analysis for Biomarker discovery using Latent Variables
***ECO***: Organic/eco-friendly housing system
***FDR***: False discovery rate
***G×E***: Genotype-by-environment interaction
***GL***: German Landrace pig
***GS***: German Saddleback pig
***GIT***: Gastrointestinal tract
***mRNA***: Messenger RNA
***OTU***: Operational taxonomic unit
***PCA***: Principal component analysis
***PCoA***: Principal coordinates analysis
***PERMANOVA***: Permutational multivariate analysis of variance
***RIN***: RNA Integrity Number
***RNA-seq***: RNA sequencing
***SVA***: Surrogate variable analysis
***VST***: Variance-stabilizing transformation

5. **References**

1.	Leenhouwers J, Merks J: **Suitability of traditional and conventional pig breeds in organic and low-input production systems in Europe: Survey results and a review of literature**. *Animal Genetic Resources/Resources génétiques animales/Recursos genéticos animales* 2013, **53**:169-184.

2.	Nürnberg K, Kuhn G, Ender K, Nürnberg G, Hartung M: **Characteristics of carcass composition, fat metabolism and meat quality of genetically different pigs**. *Lipid/Fett* 1997, **99**(12):443-446.

3.	Oster M, Gladbach C, Vernunft A, Reyer H, Otten W, Metges C, Muráni E, Ponsuksili S, Frölich K, Wimmers K: **Influence of genotype-environment interaction on stress parameters during spontaneous farrowing in modern and traditional pig breeds housed in crates and pens**. *Theriogenology* 2025, **240**:117394.

4.	Olschewsky A, Krieger M, Hoischen-Taubner S, Mörlein D, Klingel S, Thaller G, Hinrichs D: **Performance, carcass quality and meat quality of the endangered German Angler Saddleback pig**. *Plos one* 2024, **19**(4):e0300361.

5.	Werner DN: **Genotyp-Umwelt-Interaktionen bei der Haltung unterschiedlicher genetischer Schweineherkünfte unter Konventionellen und Ökologischenbedingunge; Mastleistung, Schlachtkörperqualität und Fleischzusammensetzung**. *Dissertation Justus-Liebig-Universität Gießen* 2009(VVB LAUFERSWEILER VERLAG, Giessen. Printed in Germany).

6.	Brandt H, Werner D, Gruber S, Baulain U, Henning M, Brade W, Weißmann F, Fischer K: **Evaluation of tissue growth performance, carcass quality, meat quality and breeding suitability of different pig genotypes for organic pork production**. 2009:BÖL-Projekt 03 OE 323: Universität Gießen.

7.	Guy J, Rowlinson P, Chadwick J, Ellis M: **Health conditions of two genotypes of growing-finishing pig in three different housing systems: implications for welfare**. *Livestock Production Science* 2002, **75**(3):233-243.

8.	Besteiro R, Arango T, Rodríguez MR, Fernández MD: **Linear and Nonlinear Mixed Models to Determine the Growth Curves of Weaned Piglets and the Effect of Sex on Growth**. *Agriculture* 2023, **14**(1):79.

9.	Kijlstra A, Jongert E: **Control of the risk of human toxoplasmosis transmitted by meat**. *International journal for parasitology* 2008, **38**(12):1359-1370.

10.	Papatsiros V, Athanasiou LV, Stougiou D, Christodoulopoulos G, Boutsini S: **Trichinella britovi as a risk factor for alternative pig production systems in Greece and Europe**. *Veterinary research forum : an international quarterly journal* 2020, **11**(3):199-205.

11.	Hu J, Chen J, Ma L, Hou Q, Zhang Y, Kong X, Huang X, Tang Z, Wei H, Wang X: **Characterizing core microbiota and regulatory functions of the pig gut microbiome**. *The ISME Journal* 2024, **18**(1):wrad037.

12.	Luo Y, Ren W, Smidt H, Wright A-DG, Yu B, Schyns G, McCormack UM, Cowieson AJ, Yu J, He J: **Dynamic distribution of gut microbiota in pigs at different growth stages: composition and contribution**. *Microbiology spectrum* 2022, **10**(3):e00688-00621.

13.	Meng Q, Luo Z, Cao C, Sun S, Ma Q, Li Z, Shi B, Shan A: **Weaning alters intestinal gene expression involved in nutrient metabolism by shaping gut microbiota in pigs**. *Frontiers in microbiology* 2020, **11**:694.

14.	Fouhse J, Zijlstra R, Willing B: **The role of gut microbiota in the health and disease of pigs**. *Animal Frontiers* 2016, **6**(3):30-36.

15.	Gresse R, Chaucheyras-Durand F, Fleury MA, Van de Wiele T, Forano E, Blanquet-Diot S: **Gut microbiota dysbiosis in postweaning piglets: understanding the keys to health**. *Trends in microbiology* 2017, **25**(10):851-873.

16.	He Y, Li Y, Pan Y, Li A, Huang Y, Mi Q, Zhao S, Zhang C, Ran J, Hu H *et al*: **Correlation analysis between jejunum metabolites and immune function in Saba and Landrace piglets**. *Frontiers in veterinary science* 2023, **10**:1069809.

17.	Camarinha-Silva A, Maushammer M, Wellmann R, Vital M, Preuss S, Bennewitz J: **Host genome influence on gut microbial composition and microbial prediction of complex traits in pigs**. *Genetics* 2017, **206**(3):1637-1644.

18.	Lührmann A, Ovadenko K, Hellmich J, Sudendey C, Belik V, Zentek J, Vahjen W: **Characterization of the fecal microbiota of sows and their offspring from German commercial pig farms**. *PLoS One* 2021, **16**(8):e0256112.

19.	Bergamaschi M, Tiezzi F, Howard J, Huang YJ, Gray KA, Schillebeeckx C, McNulty NP, Maltecca C: **Gut microbiome composition differences among breeds impact feed efficiency in swine**. *Microbiome* 2020, **8**(1):110.

20.	Upadhaya SD, Kim IH: **Maintenance of gut microbiome stability for optimum intestinal health in pigs - a review**. *J Anim Sci Biotechnol* 2022, **13**(1):140.

21.	Gaio D, DeMaere MZ, Anantanawat K, Chapman TA, Djordjevic SP, Darling AE: **Post-weaning shifts in microbiome composition and metabolism revealed by over 25 000 pig gut metagenome-assembled genomes**. *Microbial genomics* 2021, **7**(8).

22.	Grześkowiak ŁM, Pieper R, Huynh HA, Cutting SM, Vahjen W, Zentek J: **Impact of early-life events on the susceptibility to Clostridium difficile colonisation and infection in the offspring of the pig**. *Gut Microbes* 2019, **10**(2):251-259.

23.	Sarpong N, Seifert J, Bennewitz J, Rodehutscord M, Camarinha-Silva A: **Microbial signatures and enterotype clusters in fattening pigs: implications for nitrogen utilization efficiency**. *Frontiers in Microbiology* 2024, **15**:1354537.

24.	Kapoor K, Oster M, Reyer H, Metges CC, Muráni E, Ponsuksili S, Wimmers K: **Haematopoietic Traits in Indigenous and Modern Pig Breeds Are Modulated by Housing System and Developmental Stage**. *Animal Genetics* 2026, **57**(2):e70103.

25.	Brown J, Pirrung M, McCue LA: **FQC Dashboard: integrates FastQC results into a web-based, interactive, and extensible FASTQ quality control tool**. *Bioinformatics* 2017, **33**(19):3137-3139.

26.	Krueger F: **Trim Galore!: A wrapper around Cutadapt and FastQC to consistently apply adapter and quality trimming to FastQ files, with extra functionality for RRBS data**. *Babraham Institute* 2015.

27.	Kim D, Paggi JM, Park C, Bennett C, Salzberg SL: **Graph-based genome alignment and genotyping with HISAT2 and HISAT-genotype**. *Nature biotechnology* 2019, **37**(8):907-915.

28.	Anders S, Pyl PT, Huber W: **HTSeq—a Python framework to work with high-throughput sequencing data**. *bioinformatics* 2015, **31**(2):166-169.

29.	Leek JT, Johnson WE, Parker HS, Jaffe AE, Storey JD: **The sva package for removing batch effects and other unwanted variation in high-throughput experiments**. *Bioinformatics* 2012, **28**(6):882-883.

30.	Love MI, Huber W, Anders S: **Moderated estimation of fold change and dispersion for RNA-seq data with DESeq2**. *Genome biology* 2014, **15**(12):550.

31.	Wickham H: **ggplot2**. *Wiley interdisciplinary reviews: computational statistics* 2011, **3**(2):180-185.

32.	Dennis Jr G, Sherman BT, Hosack DA, Yang J, Gao W, Lane HC, Lempicki RA: **DAVID: database for annotation, visualization, and integrated discovery**. *Genome biology* 2003, **4**(9):R60.

33.	Bindea G, Mlecnik B, Hackl H, Charoentong P, Tosolini M, Kirilovsky A, Fridman W-H, Pagès F, Trajanoski Z, Galon J: **ClueGO: a Cytoscape plug-in to decipher functionally grouped gene ontology and pathway annotation networks**. *Bioinformatics* 2009, **25**(8):1091-1093.

34.	Bindea G, Galon J, Mlecnik B: **CluePedia Cytoscape plugin: pathway insights using integrated experimental and in silico data**. *Bioinformatics* 2013, **29**(5):661-663.

35.	Shannon P, Markiel A, Ozier O, Baliga NS, Wang JT, Ramage D, Amin N, Schwikowski B, Ideker T: **Cytoscape: a software environment for integrated models of biomolecular interaction networks**. *Genome research* 2003, **13**(11):2498-2504.

36.	Hugerth LW, Wefer HA, Lundin S, Jakobsson HE, Lindberg M, Rodin S, Engstrand L, Andersson AF: **DegePrime, a program for degenerate primer design for broad-taxonomic-range PCR in microbial ecology studies**. *Applied and environmental microbiology* 2014, **80**(16):5116-5123.

37.	Schloss PD, Westcott SL, Ryabin T, Hall JR, Hartmann M, Hollister EB, Lesniewski RA, Oakley BB, Parks DH, Robinson CJ: **Introducing mothur: open-source, platform-independent, community-supported software for describing and comparing microbial communities**. *Applied and environmental microbiology* 2009, **75**(23):7537-7541.

38.	McMurdie PJ, Holmes S: **phyloseq: an R package for reproducible interactive analysis and graphics of microbiome census data**. *PloS one* 2013, **8**(4):e61217.

39.	Oksanen J, Blanchet FG, Kindt R, Legendre P, Minchin PR, O’hara R, Simpson GL, Solymos P, Stevens MHH, Wagner H: **Package ‘vegan’**. *Community ecology package, version* 2013, **2**(9):1-295.

40.	Rohart F, Gautier B, Singh A, Lê Cao K-A: **mixOmics: An R package for ‘omics feature selection and multiple data integration**. *PLoS computational biology* 2017, **13**(11):e1005752.

41.	Stoll B, Henry J, Reeds PJ, Yu H, Jahoor F, Burrin DG: **Catabolism dominates the first-pass intestinal metabolism of dietary essential amino acids in milk protein-fed piglets**. *The Journal of nutrition* 1998, **128**(3):606-614.

42.	Guerbette T, Boudry G, Lan A: **Mitochondrial function in intestinal epithelium homeostasis and modulation in diet-induced obesity**. *Molecular metabolism* 2022, **63**:101546.

43.	Bekebrede AF, de Boer VC, Gerrits WJ, Keijer J: **Functional and molecular profiling of fasted piglets reveals decreased energy metabolic function and cell proliferation in the small intestine**. *American Journal of Physiology-Gastrointestinal and Liver Physiology* 2023, **325**(6):G539-G555.

44.	Wang C, Cao S, Shen Z, Hong Q, Feng J, Peng Y, Hu C: **Effects of dietary tributyrin on intestinal mucosa development, mitochondrial function and AMPK-mTOR pathway in weaned pigs**. *Journal of Animal Science and Biotechnology* 2019, **10**(1):93.

45.	Zhang S, Xiang Y, Jian Y, Zhao Q, Sun J, Huang Y, Xu J, Qi X, Li J, Zheng Z: **Uncovering Molecular Mechanisms of Feed Efficiency in Pigs Through Multi-Omics Analysis of the Jejunum**. *Animals: an Open Access Journal from MDPI* 2025, **15**(2):137.

46.	Sun X, Yang Q, Rogers CJ, Du M, Zhu M-J: **AMPK improves gut epithelial differentiation and barrier function via regulating Cdx2 expression**. *Cell Death & Differentiation* 2017, **24**(5):819-831.

47.	Kumar ST, Zheng Y, Xu J, Zhao Z, Zhang Q, Zhang Y, Li M, Zou H, Azeem RM, Sun WS *et al*: **Transcriptome and Metabolome Insights into Key Genes Regulating Fat Deposition and Meat Quality in Pig Breeds**. *Animals : an open access journal from MDPI* 2024, **14**(24).

48.	Lickfett H, Oster M, Vernunft A, Reyer H, Görs S, Metges C, Bostedt H, Wimmers K: **Influence of genotype and environment on piglet vitality and metabolic state during the suckling period of divergent birth weight classes**. *animal* 2025, **19**(4):101460.

49.	Kumar ST, Zhang Y, Zhang Q, Azeem RM, Jing Z, Pan L, Sun WS, Zhao Y, Zhang SM: **Breed-specific differences of gut microbiota and metabolomic insights into fat deposition and meat quality in Chinese Songliao Black Pig and Large White × Landrace Pig Breeds**. *BMC Microbiol* 2025, **25**(1):334.

50.	Zhang Y, Sun L, Zhu R, Zhang S, Liu S, Wang Y, Wu Y, Xing S, Liao X, Mi J: **Porcine gut microbiota in mediating host metabolic adaptation to cold stress**. *npj Biofilms and Microbiomes* 2022, **8**(1):18.

51.	Singh V, Lee G, Son H, Koh H, Kim ES, Unno T, Shin J-H: **Butyrate producers,“The Sentinel of Gut”: Their intestinal significance with and beyond butyrate, and prospective use as microbial therapeutics**. *Frontiers in microbiology* 2023, **13**:1103836.

52.	Fitzgerald BG, Govind M, Firth IJ, Herold L, Froment J, Vancuren SJ, Allen-Vercoe E, Kimber MS, Sorbara MT: **Members of Lachnospiraceae produce valerate and caproate in response to short-chain fatty acids**. *Microbiome* 2025, **13**(1):201.

53.	Azouggagh L, Ibáñez-Escriche N, Martínez-Álvaro M, Varona L, Casellas J, Negro S, Casto-Rebollo C: **Characterization of microbiota signatures in Iberian pig strains using machine learning algorithms**. *Animal Microbiome* 2025, **7**(1):13.

54.	Arsenault M, Lillie B, Nadeem K, Khafipour E, Farzan A: **Progression of swine fecal microbiota during early stages of life and its association with performance: a longitudinal study**. *BMC microbiology* 2024, **24**(1):182.

55.	Kavanova K, Kostovova I, Moravkova M, Kubasova T, Crhanova M: **In vitro characterization of lactic acid bacteria and bifidobacteria from wild and domestic pigs: probiotic potential for post-weaning piglets**. *BMC microbiology* 2025, **25**(1):8.

56.	Wedemeyer J, Lechleiter N, Vernunft A, Junker J, Homeier-Bachmann T: **Impact of Organic and Conventional Husbandry Systems on the Gut Microbiome and Resistome in Pigs**. *Microorganisms* 2025, **13**(9):2161.

57.	Wang J, Zhu L, Wang Y, Ma Q, Yan X, Li M, Xing B: **Impact of the Gut Microbiota–Metabolite Axis on Intestinal Fatty Acid Absorption in Huainan Pigs**. *Microorganisms* 2025, **13**(7):1609.

58.	Holman DB, Gzyl KE, Kommadath A: **The gut microbiome and resistome of conventionally vs. pasture-raised pigs**. *Microbial Genomics* 2023, **9**(7):001061.

59.	Zhou H, Yu B, Sun J, Liu Z, Chen H, Ge L, Chen D: **Short-chain fatty acids can improve lipid and glucose metabolism independently of the pig gut microbiota**. *Journal of animal science and biotechnology* 2021, **12**(1):61.

60.	Saladrigas-García M, D’Angelo M, Ko H-L, Traserra S, Nolis P, Ramayo-Caldas Y, Folch JM, Vergara P, Llonch P, Pérez JF: **Early socialization and environmental enrichment of lactating piglets affects the caecal microbiota and metabolomic response after weaning**. *Scientific reports* 2021, **11**(1):6113.

61.	Wen C, Van Dixhoorn I, Schokker D, Woelders H, Stockhofe-Zurwieden N, Rebel JM, Smidt H: **Environmentally enriched housing conditions affect pig welfare, immune system and gut microbiota in early life**. *Animal Microbiome* 2021, **3**(1):52.

62.	Stepanova H, Scheirichova M, Matiasovic J, Hlavova K, Sinkora M, Stepanova K, Faldyna M: **Interleukin-17 producing cells in swine induced by microbiota during the early postnatal period-a brief research report**. *Frontiers in Immunology* 2023, **14**:1214444.

63.	Cerezo EL, Henry Y, Henras AK, Romeo Y: **Ribosome biogenesis and homeostasis: in the front line to cope with cellular stress**. *Biochimie* 2025.

64.	Jobava R, Mao Y, Guan B-J, Hu D, Krokowski D, Chen C-W, Shu XE, Chukwurah E, Wu J, Gao Z: **Adaptive translational pausing is a hallmark of the cellular response to severe environmental stress**. *Molecular cell* 2021, **81**(20):4191-4208. e4198.

65.	Vigors S, Sweeney T, O’shea C, Kelly A, O’doherty J: **Pigs that are divergent in feed efficiency, differ in intestinal enzyme and nutrient transporter gene expression, nutrient digestibility and microbial activity**. *Animal* 2016, **10**(11):1848-1855.

66.	Cheng Y, Ding S, Azad MAK, Song B, Kong X: **Comparison of the pig breeds in the small intestinal morphology and digestive functions at different ages**. *Metabolites* 2023, **13**(1):132.

67.	Wang Z, Hu J, Zheng W, Yang T, Wang X, Xie C, Yan X: **Lactobacillus frumenti mediates energy production via fatty acid β-oxidation in the liver of early-weaned piglets**. *Journal of Animal Science and Biotechnology* 2019, **10**(1):95.

68.	Wang W, Zijlstra RT, Gänzle MG: **Feeding Limosilactobacillus fermentum K9-2 and Lacticaseibacillus casei K9-1, or Limosilactobacillus reuteri TMW1.656 Reduces Pathogen Load in Weanling Pigs**. *Front Microbiol* 2020, **11**:608293.

69.	Oh JK, Vasquez R, Kim SH, Hwang I-C, Song JH, Park JH, Kim IH, Kang D-K: **Multispecies probiotics alter fecal short-chain fatty acids and lactate levels in weaned pigs by modulating gut microbiota**. *Journal of animal science and technology* 2021, **63**(5):1142.

70.	Denis M, Nagard B, Rose V, Bourgoin K, Cutimbo M, Kerouanton A: **No Clear Differences between Organic or Conventional Pig Farms in the Genetic Diversity or Virulence of Campylobacter coli Isolates**. *Frontiers in Microbiology* 2017, **Volume 8 - 2017**.

71.	Kempf I, Kerouanton A, Bougeard S, Nagard B, Rose V, Mourand G, Osterberg J, Denis M, Bengtsson BO: **Campylobacter coli in Organic and Conventional Pig Production in France and Sweden: Prevalence and Antimicrobial Resistance**. *Front Microbiol* 2017, **8**:955.

72.	Rath A, Rautenschlein S, Rzeznitzeck J, Lalk M, Methling K, Rychlik I, Peh E, Kittler S, Waldmann KH, von Altrock A: **Investigation on the colonisation of Campylobacter strains in the pig intestine depending on available metabolites**. *Comp Immunol Microbiol Infect Dis* 2022, **88**:101865.

73.	Kulathunga DGRS, Fakher AA, Costa MdO: **Actinobacillus suis isolated from diseased pigs are phylogenetically related but harbour different number of toxin gene copies in their genomes**. *Veterinary Record Open* 2022, **9**(1):e45.

74.	Fredriksen S, Neila-Ibáñez C, Hennig-Pauka I, Guan X, Dunkelberger J, de Oliveira IF, Ferrando ML, Correa-Fiz F, Aragon V, Boekhorst J: **Streptococcus suis infection on European farms is associated with an altered tonsil microbiome and resistome**. *Microbial Genomics* 2024, **10**(12):001334.

75.	Neila-Ibáñez C, Casal J, Hennig-Pauka I, Stockhofe-Zurwieden N, Gottschalk M, Migura-García L, Pailler-García L, Napp S: **Stochastic assessment of the economic impact of Streptococcus suis-associated disease in German, Dutch and Spanish swine farms**. *Frontiers in veterinary science* 2021, **8**:676002.

76.	Li C, Wang Y, Zhao X, Li J, Wang H, Ren Y, Sun H, Zhu X, Song Q, Wang J: **Comparative analysis of intestinal inflammation and microbiota dysbiosis of LPS-challenged piglets between different breeds**. *Animals* 2024, **14**(5):665.

77.	Jinno C, Kim K, Wong B, Wall E, Sripathy R, Liu Y: **Dietary Supplementation with Botanical Blends Modified Intestinal Microbiota and Metabolomics of Weaned Pigs Experimentally Infected with Enterotoxigenic Escherichia coli**. *Microorganisms* 2023, **11**(2):320.

78.	Alt K, Fetsch A, Schroeter A, Guerra B, Hammerl JA, Hertwig S, Senkov N, Geinets A, Mueller-Graf C, Braeunig J: **Factors associated with the occurrence of MRSA CC398 in herds of fattening pigs in Germany**. *BMC veterinary research* 2011, **7**(1):69.

79.	Bäckhed F, Ding H, Wang T, Hooper LV, Koh GY, Nagy A, Semenkovich CF, Gordon JI: **The gut microbiota as an environmental factor that regulates fat storage**. *Proc Natl Acad Sci U S A* 2004, **101**(44):15718-15723.

80.	Wahlström A, Sayin SI, Marschall HU, Bäckhed F: **Intestinal Crosstalk between Bile Acids and Microbiota and Its Impact on Host Metabolism**. *Cell Metab* 2016, **24**(1):41-50.

81.	Richards AL, Muehlbauer AL, Alazizi A, Burns MB, Findley A, Messina F, Gould TJ, Cascardo C, Pique-Regi R, Blekhman R *et al*: **Gut Microbiota Has a Widespread and Modifiable Effect on Host Gene Regulation**. *mSystems* 2019, **4**(5).

82.	Levy E, Ben Djoudi Ouadda A, Spahis S, Sane AT, Garofalo C, Grenier É, Emonnot L, Yara S, Couture P, Beaulieu JF *et al*: **PCSK9 plays a significant role in cholesterol homeostasis and lipid transport in intestinal epithelial cells**. *Atherosclerosis* 2013, **227**(2):297-306.

83.	Rollo SN, Norby B, Bartlett PC, Scott HM, Wilson DL, Fajt VR, Linz JE, Bunner CE, Kaneene JB, Huber JC, Jr.: **Prevalence and patterns of antimicrobial resistance in Campylobacter spp isolated from pigs reared under antimicrobial-free and conventional production methods in eight states in the Midwestern United States**. *J Am Vet Med Assoc* 2010, **236**(2):201-210.

84.	Ager EO, Carvalho T, Silva EM, Ricke SC, Hite JL: **Global trends in antimicrobial resistance on organic and conventional farms**. *Sci Rep* 2023, **13**(1):22608.

85.	O’Flaherty S, Briner Crawley A, Theriot CM, Barrangou R: **The Lactobacillus bile salt hydrolase repertoire reveals niche-specific adaptation**. *MSphere* 2018, **3**(3):10.1128/msphere. 00140-00118.

86.	Lin J, Sahin O, Michel LO, Zhang Q: **Critical role of multidrug efflux pump CmeABC in bile resistance and in vivo colonization of Campylobacter jejuni**. *Infection and immunity* 2003, **71**(8):4250-4259.

87.	Shimizu T: **Inflammation-inducing Factors of Mycoplasma pneumoniae**. *Front Microbiol* 2016, **7**:414.

**Acknowledgments**

The authors thank Birgit Mielenz, Leon Borowski, Nicole Gentz, Anna Zenk, Joana Bittner, and Anja Reyer for their excellent technical assistance. We greatly appreciate the work of the staff at the Experimental Pig Facility, particularly Marianne Zenk, as well as the team at the slaughterhouse of the Research Institute for Farm Animal Biology (FBN). We also thank Shuaichen Li, Avon Augustin Nalpadan and Alayu Kidane Solomon for their valuable assistance with gut sample collection. Finally, we thank all colleagues involved in the initiation and establishment of the Genotype-by-Environment Interactions (G×E) experiment.

**Funding**

### This research received no external funding.

### **Authors’ contributions**

### **Conceptualization, Klaus Wimmers, and Siriluck Ponsuksili; Data curation, Henry Reyer, Nares Trakooljul; Formal analysis, Hina Wahab Khan,** **Muhammad Arsalan Iqbal, and Henry Reyer; Investigation, Hina Wahab Khan, Nares Trakooljul, Michael Oster and Henry Reyer; Methodology,** **Bjoern Kuhla, Jonas Keiler, Henry Reyer, Siriluck Ponsuksili; Software, Henry Reyer, Hina Wahab Khan, Muhammad Arsalan Iqbal and Frieder Hadlich; Supervision,** **Siriluck Ponsuksili; Writing – original draft, Hina Wahab Khan,** **Muhammad Arsalan Iqbal and Siriluck Ponsuksili; Writing – review & editing, Bjoern Kuhla, Jonas Keiler, Michael Oster, Henry Reyer, Nares Trakooljul, Klaus Wimmers, and Siriluck Ponsuksili. All authors reviewed and approved the manuscript.**

### **Ethics Declarations**

**Ethics approval and consent to participate**

All experiments were performed in accordance with the German Animal Welfare Act, and EU Directive 2010/63/EU (Annex VIII, Section III). The study complied with the ARRIVE guidelines and received ethical approval from the Ethics Committee of the federal state of Mecklenburg-Western Pomerania, Germany (LALLF M-V/TSD/7221.3-1-030/21).

**Clinical trial number:** Not applicable.

**Consent for publication**
Not applicable.

**Competing interests**
The authors declare that they have no competing interests.

**Supplementary Information**

Supplementary materials for this study include nine tables.

**Supplementary Table S1.** Nutrient composition of piglet diets at the pre-starter, starter/growing, and finishing stages.

**Supplementary Table S2.** Mapping statistics of mRNA reads for all RNA-seq libraries.

**Supplementary Table S3.** Differential gene expression statistics for main effects (breed, housing system, and sex).

**Supplementary Table S4.** Functional enrichment of DEGs associated with main effects (breed and housing system).

**Supplementary Table S5.** Differential gene expression statistics for the combined group effect.

**Supplementary Table S6.** Functional enrichment of DEGs associated with the combined group effect.

**Supplementary Table S7.** Alpha and beta diversity statistics of jejunal digesta microbiota.

**Supplementary Table S8.** Differentially abundant microbial genera across main effects (breed, housing system) and the combined group effect.

**Supplementary Table S9.** Univariate DESeq2 statistics for DIABLO-selected features across breed–housing group contrasts.
